# Diverticulosis and cardiometabolic risk factors: a systematic literature review

**DOI:** 10.1007/s00384-023-04532-4

**Published:** 2023-09-19

**Authors:** Andreas Völkerer, Sarah Wernly, Georg Semmler, Maria Flamm, Elmar Aigner, Christian Datz, Bernhard Wernly

**Affiliations:** 1 Department of Internal Medicine, General Hospital Oberndorf, Teaching Hospital of the Paracelsus Medical University, Salzburg, Austria; 2 Division of Gastroenterology and Hepatology, Department of Medicine III, Medical University of Vienna, Vienna, Austria; 3 Institute of General Practice, Family Medicine and Preventive Medicine, Paracelsus Medical University, Strubergasse 21, 5020 Salzburg, Austria; 4 Clinic I for Internal Medicine, University Hospital Salzburg, Paracelsus Medical University, Salzburg, Austria

**Keywords:** Diverticulosis, Metabolic syndrome, Obesity, Arterial hypertension, Diabetes

## Abstract

**Background:**

There is a hypothesis of an association between diverticulosis and metabolic syndrome (MS) or its components, but data on this topic are inconsistent, and a systematic review has not been performed. We conducted a systematic review to investigate the possible association between cardiometabolic risk factors and diverticulosis.

**Methods:**

A systematic literature search was conducted via PubMed, Cochrane Library, and Web of Science in December 2022 to collect the necessary data. Studies that examined the association between MS or individual metabolic factors and asymptomatic diverticulosis were included in the review.

**Results:**

Of the potentially relevant articles identified via PubMed (477), Cochrane Library (224), and Web of Science (296), 29 articles met the inclusion criteria and were used for this work. These studies were assessed for study quality using GRADE. Overall, 6 studies were rated as “very low,” 19 studies as “low,” and 4 studies as “moderate.” The data suggest an association between arterial hypertension, obesity, and fatty liver disease in younger patients and diverticulosis. Patient age appears to play an important role in diverticular formation. Data on diabetes mellitus is inconclusive and may require further investigation depending on the location of the diverticula.

**Conclusion:**

Based on the synthesized data, there is an association between arterial hypertension, obesity, and fatty liver disease in younger patients. The formation of diverticula seems to be influenced by age and genetic factors. The study suggests a connection with cardiometabolic risk factors. To gain a better understanding of the role of metabolic risk factors in asymptomatic diverticulosis, targeted studies are necessary based on these findings.

## Introduction

Diverticulosis is a condition characterized by the presence of small protrusions in the intestinal wall, which are called diverticula. These protrusions can impact various layers of the intestinal wall depending on their location and are among the most frequently observed alterations in the colon. In the left segment of the colon, this change causes the herniation of the mucosa and submucosa through the muscular layer. These protrusions occur at the weakest points of the muscular layer, where blood vessels penetrate. These changes are referred to as pseudodiverticula because only specific layers of the intestinal wall are affected in this case. On the other hand, in the right colonic segment, all layers of the wall are more commonly affected, classifying them as true diverticula [1]. The location of these diverticular formations varies according to ancestry. In the Western world, diverticula are predominantly found in individuals of white ethnicity, with a higher prevalence in the left colon (86%), particularly in the sigmoid colon [2]. In contrast, individuals of Asian descent primarily develop diverticula in the right colon, regardless of sex, age, or ethnicity, even in highly Westernized regions [3–5]. Diverticulosis becomes more prevalent as people age, affecting approximately one-third of patients under 60 and around 70% of those over 80 [6]. Although diverticulosis is a common condition, only 4% of those affected develop acute diverticulitis, which is characterized by inflammation of the diverticula. Younger individuals have a higher risk of developing this disease [7]. In addition to the growing prevalence of diverticulosis, there has been a 50% rise in the occurrence of diverticulitis in recent years [8]. Several potential risk factors for the development of these diverticula have been discussed. In addition to rare genetic syndromes such as Marfan syndrome [9, 10], Ehlers-Danlos syndrome [10, 11], Williams-Beuren syndrome [12], Coffin-Lowry syndrome [13], and polycystic kidney disease [10], which are associated with defects in the extracellular matrix and connective tissue structures, a genetic predisposition is a decisive factor in the development of these diverticula [14]. Besides genetics, dietary habits and environmental factors also contribute to the development of asymptomatic diverticula. However, the data pertaining to this matter is inconclusive. Contrary to earlier assumptions that a high-fiber diet could protect against the development of diverticula, recent evidence suggests that there is no apparent link between a high-fiber diet and the occurrence of diverticula [15, 16]. Despite popular belief, constipation is not a risk factor for the formation of diverticula [16].

In addition to ethnic, bio-chronological, and dietary factors, lifestyle factors, particularly cardiovascular and metabolic risk factors, have also been linked to the development of diverticula. The association between cardiovascular and metabolic risk factors and diverticula is of interest due to the potential for a reciprocal relationship, wherein diverticulosis can impact cardiovascular and metabolic outcomes, and vice versa. The accumulation of diverticula formations has been reported in hypertensive patients [17], as well as in individuals with increased alcohol consumption, high-fat diet [18], and obesity [19]. However, no systematic review has been conducted on this topic. The objective of this systematic review is to offer a comprehensive overview of the heterogeneous data and the presumed correlation between diverticulosis and cardiometabolic risk factors.

## Methods

A systematic literature search was performed using the PubMed, Cochrane Library, and Web of Science databases in December 2022. Applying the search strategy including the terms “diverticular disease” OR “diverticulosis” AND (“metabolic syndrome” OR “metabolic dysfunction” OR obesity OR “arterial hypertension” OR “diabetes” OR “hyperlipidaemia” OR “hypercholesterolaemia” OR “NAFLD” OR “MAFLD”), 477 studies between May 1952 and December 2022 were found via PubMed. Under the same search conditions, Web of Science provided 296 studies, and the Cochrane Library provided 224 studies. The systematic literature search was performed by two independent researchers (B.W. and A.V.) using the described search strategy. No language restriction was applied.

### PICO

We used the PECO scheme for our systematic review of observational studies [20].

- Patients: all individuals screened for diverticulosis by any means
- Exposure: presence of diverticulosis or diverticular disease
- Control: absence of diverticulosis
- Outcome: presence of metabolic syndrome or subcomponents of a dysmetabolic disorder

### Assessment of quality and risk of bias

The most recent update of the Grading of Recommendations Assessment, Development and Evaluation (GRADE) approach was employed to assess the risk of bias in the included studies [21]. The GRADE system evaluates the quality of evidence across studies and considers several factors, including study design, consistency of results, directness of evidence, precision, and publication bias, among others.

The ROBINS-E (Risk Of Bias In Non-randomized Studies—of Exposures) tool is a comprehensive assessment framework designed to evaluate the risk of bias in non-randomized studies assessing the effects of exposures. It provides a systematic approach for researchers to assess the quality and reliability of evidence from such studies, helping to ensure that the results are robust and can inform decision-making [22].

Two independent researchers (B.W. and A.V.) were responsible for conducting the quality and risk of bias assessment. Discrepancies that arose between the researchers regarding the evaluation of any study were addressed through a consensus-driven process.

### Eligibility criteria

Case-control studies, cross-sectional studies, and cohort studies examining the association between diverticulosis and metabolic syndrome or subcomponents of a dysmetabolic disorder were included in this systematic literature search. A prerequisite for inclusion was an available effect assessment using OR, RR, HR, or standardized incidence rates with 95% CI. Screening for inclusion in the study was performed independently by the two investigators mentioned above and subsequently reevaluated in conference. The definition of the diagnosis of a respective metabolic component or MS in the selected studies was considered in the interpretation. Studies were excluded if the inclusion criteria were not met or if the studies had not been performed in humans. Review articles, editorial comments, or conference proceedings were excluded.

### Data preparation

Using a structured data collection table, the following information was extracted from the primary included studies: study title, publication year, authors, country, results, patient numbers, sex, age, and study type. Depending on availability, adjusted ORs with 95% CI were listed under results. Otherwise, only significant correlation was documented. To ensure data quality, data were reevaluated by a second investigator (B.W.) after collection by the principal investigator (A.V.).

### Statistical analysis

Due to the heterogeneity of the data and different study endpoints, this systematic literature review is a descriptive summary of the included studies. A statistical summary was considered impractical and was not performed for this reason. This study was registered online (https://osf.io/zy5wg/).

## Results

### Search and critical appraisal

The described search strategy resulted in 477 potentially relevant articles being identified with PubMed. Four hundred thirty-five of these articles were excluded after reviewing the information from their titles and abstracts, as they did not meet the inclusion criteria. The remaining 42 articles underwent full-text analysis, and 15 of them were further excluded for having inappropriate study design or endpoint. The 27 remaining articles met the inclusion criteria and were used for the current study. Web of Science provided 296 results, of which 263 could be excluded based on their titles and abstracts. After excluding an additional 8 articles through full-text analysis, there were 25 results, of which 23 matched those from the PubMed search. The 2 new articles were included in the current study. The search for relevant literature in the Cochrane Library returned 224 results, but none of them met the inclusion criteria. An additional individual literature search on UpToDate and on the included studies did not yield any further studies to include.

**Fig. 1:**
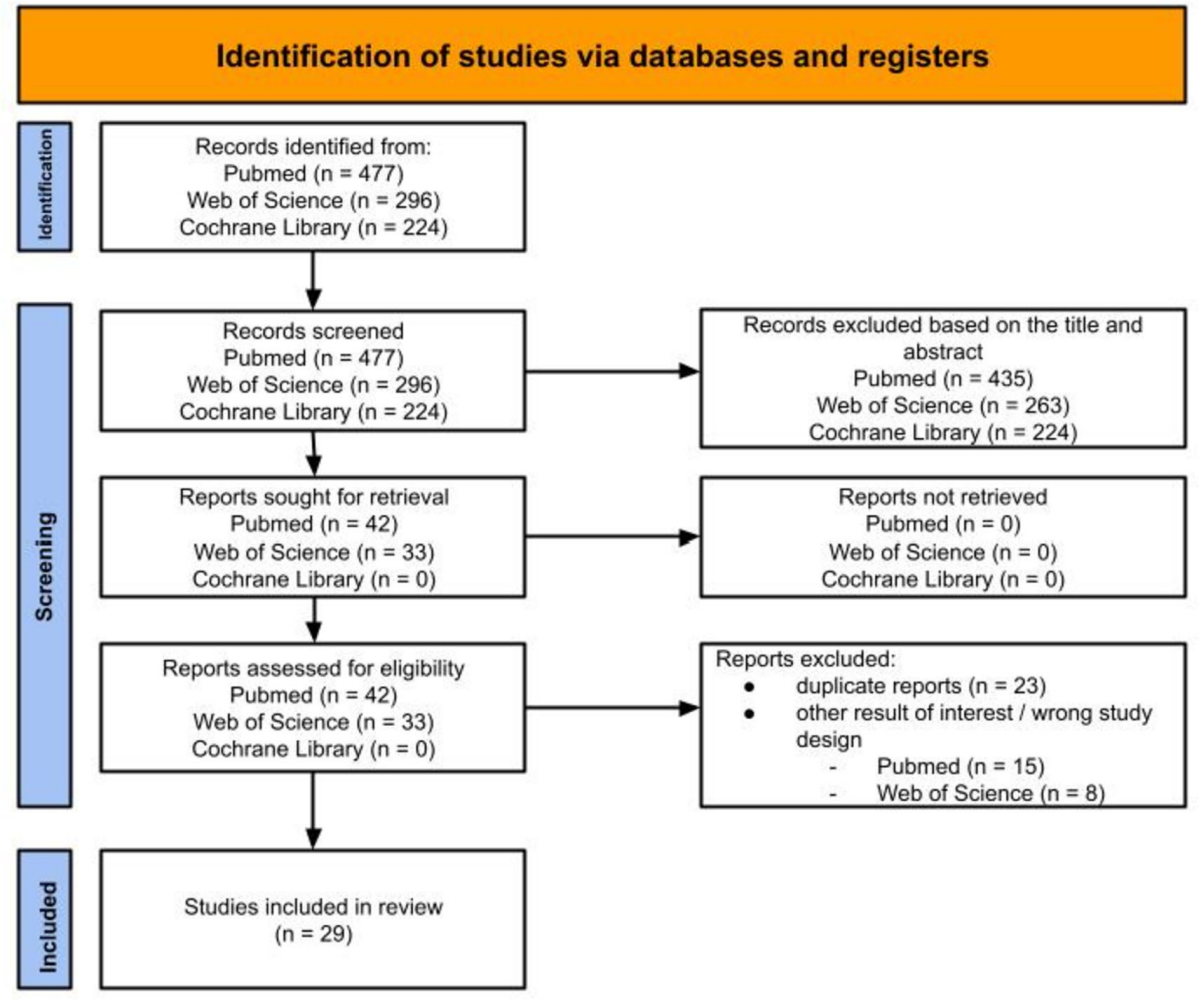
Literature review process

**Fig. 2:**
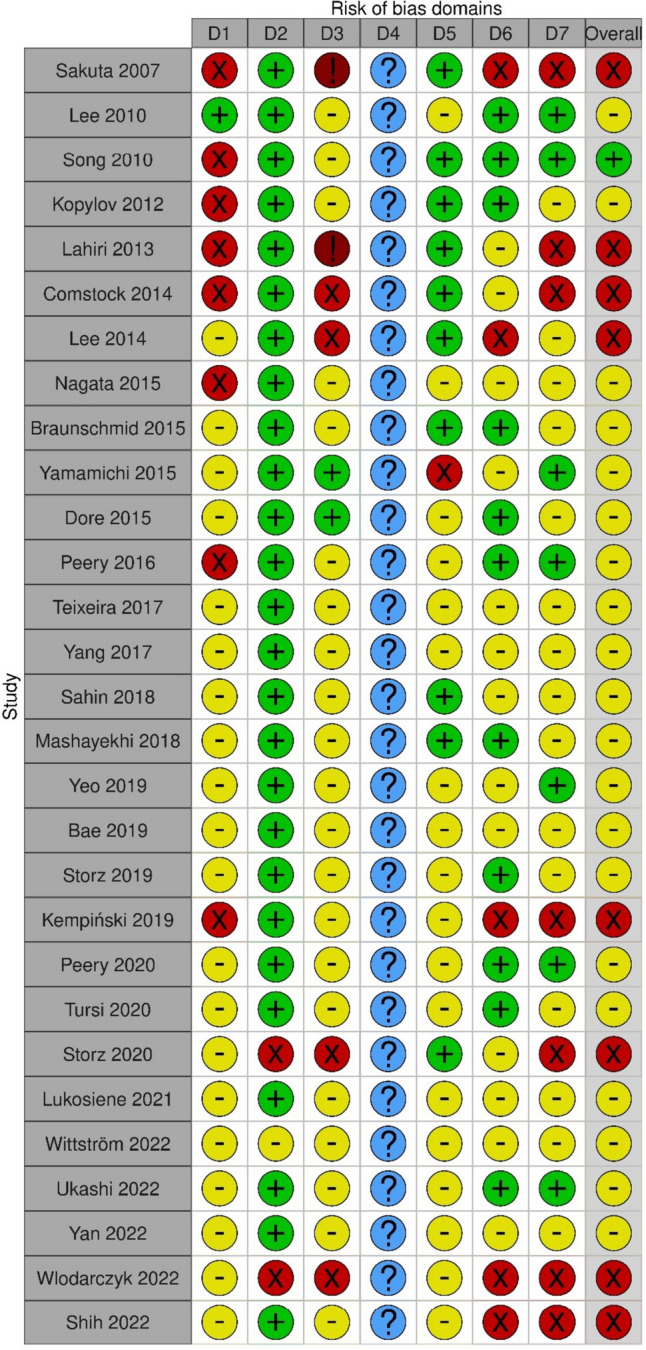
Assessment of quality and risk of bias (ROBINS-E) [22]: domain 1: risk of bias due to confounding; domain 2: risk of bias arising from measurement of the exposure; domain 3: risk of bias in selection of participants into the study (or into the analysis); domain 4: risk of bias due to post-exposure interventions; domain 5: risk of bias due to missing data; domain 6: risk of bias arising from measurement of the outcome; and domain 7: risk of bias in selection of the reported result

The literature search, review, and selection process is depicted in Fig. 1. These 29 included studies consisted of 7 prospective studies, 10 cross-sectional analyses, 9 case-control studies, one retrospective case-note review, and 2 population-based cohort studies. The quality of the studies was assessed using the GRADE method, with 6 rated as “very low,” 19 rated as “low,” and 4 rated as “moderate.” The characteristics and quality assessment of the studies are presented in Table 1. Due to the heterogeneity of the data and endpoints, the results were summarized in separate sections (Fig. 2).

**Table 1:** Selected studies

**Title**	**Year**	**Authors**	**Country**	**Exposure**	**Pts included**	**m(DIV)**	**w(DIV)**	**Age**	**Study type**	**GRADE**
Prevalence rates of type 2 diabetes and hypertension are elevated among middle-aged Japanese men with colonic diverticulum	2007	Sakuta and Suzuki [24]	Japan	Diabetes mellitus type 2 (*p* value = 0.047) and hypertension (*p* value = 0.011) elevated in middle aged subjects with DIV	954(97)	954(97)	(–)	51–59	Cross-sectional analysis	Very low
Clinical significance of colonic diverticulosis associated with bowel symptoms and colon polyp	2010	Lee et al. [23]	Korea	Age; diabetes mellitus (OR 2.08; 95% CI 1.23–3.53), polyp (OR 2.72; 95% CI 1.94–3.82) with pandiverticulosis; diabetes mellitus (OR 2.40; 95% CI 1.40–4.15), polyp (OR 2.36; 95% CI 1.66–3.38) with proximal diverticulosis	1030(203)	611(143)	419(60)	52.2	Prospective study	Low
Clinical characteristics of colonic diverticulosis in Korea: a prospective study	2010	Song et al. [18]	Korea	Age > 60 (OR 2.563; 95% CI 1.030–6.375); FAT MDA score < 6 (OR 1.763; 95% CI 1.044–2.977); alcohol (OR 2.195; 95% CI 1.091–4.416)	848(103)	518(79)	330(24)	50.9	Prospective study	Low
Obesity, metabolic syndrome and the risk of development of colonic diverticulosis	2012	Kopylov et al. [26]	Israel	Age (OR 1.070; 95% CI 1.056–1.083); male gender (OR 1.737; 95% CI 1.346–2.241); obesity (BMI > 30) (OR 1.394; 95% CI 1.099–1.769); hypothyroidism (OR 2.403; 95% CI 1.303–4.431); diabetes mellitus (OR 0.488; 95% CI 0.289–0.825)	3175(553)	2464(464)	711(89)	58.1	Retrospective case-control study	Low
A cross sectional study of colonic diverticulosis in the London Bangladeshi population	2013	Lahiri et al. [55]	Great Britain	Prevalence of diverticulosis in Bangladeshis (17/630: 2.7%) lower than Caucasians (673/1869: 36%), Indians/Pakistanis (16/161: 9.9%), Oriental (15/44: 34%), Black (90/369: 24.4%) (*p* < 0.0001 for all comparisons); significantly greater incidence of type 2 DM + ischemic heart disease (*p* < 0.0001) in Bangladeshi; less left colonic and sigmoid diverticulosis in Bangladeshi (*p* < 0.0001)	3151(905)	1510(–)	1641(–)	63.0	Cross-sectional analysis	Low
Cross-sectional analysis of obesity and serum analytes in males identifies sRAGE as a novel biomarker inversely associated with diverticulosis	2014	Comstock et al. [33]	USA	Each increased BMI category (OR 2.7; 95% CI 1.6–4.7); each increased waist circumference tertile (OR 2.8; 95% CI 1.7–4.9); each increased leptin concentration (OR 2.4; 95% CI 1.4–3.9); low molecular adiponectin (OR 0.5; 95% CI 0.3–0.8) and sRAGE (OR 0.4; 95% CI 0.3–0.7) inversely related	126(53)	126(53)	(–)	48–65	Cross-sectional analysis	Very low
The relationship between colonic diverticulosis and abdominal visceral and subcutaneous fat accumulation measured by abdominal CT scan	2014	Lee et al. [36]	Korea	Diverticulosis group: total abdominal fat area, visceral fat area, and abdominal subcutaneous fat area larger than control and diverticulitis group	133(31)	70(15)	63(16)	51.6	Retrospective case-note review	Very low
Visceral abdominal obesity measured by computed tomography is associated with increased risk of colonic diverticulosis	2015	Nagata et al. [37]	Japan	Visceral adipose tissue (VAT) and subcutaneous adipose tissue (SAT) associated (*p* for trend < 0.001) with diverticulosis, even with normal BMI (< 25); VAT positively associated with distribution: right (OR 1.79; 95% CI 1.24–2.58); left (OR 2.30; 95% CI 1.31–4.03); bilateral (OR 2.99; 95% CI 1.75–5.11)	1445(328)	949(228)	496(100)	60.7	Prospective cohort study	Low
Constipation is not associated with diverticular disease - analysis of 976 patients	2015	Braunschmid et al. [25]	Austria	Association with age (*p* < 0.0001), BMI (*p* = 0.0007), DM (*p* = 0.0178), no association with constipation (*p* = 0.1073)	976(290)	488(147)	488(143)	62.0	Prospective study	Low
Trend and risk factors of diverticulosis in Japan: age, gender, and lifestyle/metabolic-related factors may cooperatively affect on the colorectal diverticula formation	2015	Yamamichi et al. [4]	Japan	Age (OR 1.24–1.96); male (OR 1.20; 95% CI 1.08–1.35); smoking (OR 1.15–1.22); severe weight increase in adulthood (OR 1.17; 95% CI 1.06–1.28) HbA1c (OR 1.15; 95% CI 1.06–1.24) alcohol (OR 1.11; 95% CI 1.02–1.22) triglyceride (OR 1.10; 95% CI 1.02–1.20)	3327(858)	2485(734)	842(124)	55.0	Retrospective case-control study	Moderate
Risk factors associated with colonic diverticulosis among patients from a defined geographic area	2015	Dore et al. [56]	Italy	Year of birth (OR 0.974; 95% CI 0.966–0.982); cardiovascular disease (OR 1.315; 95% CI 1.041–1.661); other gastrointestinal disease (OR 1.403; 95% CI 1.132–1.739)	4458(841)	1775(386)	2683(455)	58.4/56.2	Retrospective case-control study	Low
Distribution and characteristics of colonic diverticula in a United States screening population	2016	Peery et al. [44]	USA	Age 51–60 [> 10 diverticula (OR 2.4; 95% CI 1.1–5.0); large diverticula (OR 2.6; 95% CI 1.2–5.5); deep diverticula (OR 2.8; 95% CI 1.3–6.2)]; age > 60 [> 10 diverticula (OR 5.6; 95% CI 2.5–12.6); large diverticula (OR 3.8; 95% CI 1.6–9.0); deep diverticula (OR 4.3; 95% CI 1.8–10.5)]; BMI overweight (OR 2.0; 95% CI 1.0–3.8); BMI obese (OR 3.1; 95% CI 1.7–5.8)	624(260)	274(125)	350(135)	54.0	Prospective study	Moderate
Colonic diverticulosis and the metabolic syndrome: an association?	2017	Teixeira et al. [38]	Portugal	Age (OR 1.068; 95% CI 1.027–1.111); increased waist circumference (OR 2.129, 95% CI 1.005–4.510); metabolic syndrome (OR 3.682, 95% CI 1.587–8.546)	203(62)	95(30)	108(32)	65.5	Prospective study	Low
Sex differences in risk factors of uncomplicated colonic diverticulosis in a metropolitan area from Northern China	2017	Yang et al. [57]	China	m [age (OR 1.05; 95% CI 1.03–1.06); smoking (OR 2.14; 95% CI 1.05–4.33)]; f [age (OR 1.03; 95% CI 1.01–1.05); BMI (OR 1.12; 95% CI 1.04–1.19); smoking (OR 10.2; 95% CI 2.81–37.4) hypertension (OR 1.76; 95% CI 1.01–3.06); antihypertensive medication (OR 2.99; 95% CI 1.66–5.39)]	4386(218)	2044(148)	2342(70)	52.6/54.7	Cross-sectional analysis	Low
Relationship between diverticulosis and nonalcoholic fatty liver disease in elderly patients	2018	Sahin et al. [43]	Turkey	Diverticulosis independent negative predictor of hepatosteatosis (OR 0.529; 95% CI 0.323–0.866)	355(169)	211(102)	144(67)	74.8	Retrospective case-control study	Very low
Obesity, but not physical activity, is associated with higher prevalence of asymptomatic diverticulosis	2018	Mashayekhi et al. [34]	USA	BMI 25–30 (OR 3.02; 95% CI 1.33–6.88); BMI > 30 (OR 4.43; 95% CI 1.88–10.49)	223(86)	(–)	(–)	60.8	Retrospective case-control study	Low
Hypertension control and risk of colonic diverticulosis	2019	Yeo et al. [17]	Taiwan	Hypertension (OR 1.83; 95% CI 1.21–2.75); hypertension without therapy (OR 1.73; 95% CI 1.06–2.83); hypertension despite therapy (OR 2.07, 95% CI 1.17–3.67)	2748(141)	1672(106)	1076(35)	53.21	Retrospective case-control study	Low
Risk factors for asymptomatic colon diverticulosis	2019	Bae et al. [39]	Korea	Waist-hip ratio (OR 1.035; 95% CI 1.000–1.070); moderate FLD (OR 2.238; 95% CI 1.026–4.882); severe FLD (OR 5.519; 95% CI 1.236–21.803)	937(76)	622(58)	315(18)	50.5	Retrospective case-control study	Low
Characteristics and associated risk factors of diverticular disease assessed by magnetic resonance imaging in subjects from a Western general population	2019	Storz et al. [35]	Germany	No or mild grade diverticulosis vs. advanced diverticulosis [age (OR 2.24; 95% CI 1.60–3.15); BMI (OR 1.44; 95% CI 1.06–1.96); LDL (OR 2.35; 95% CI 1.09–5.06)]	393(164)	226(98)	167(66)	56.4	Population-based cohort study	Moderate
Clinical outcomes of non-alcoholic fatty liver disease: Polish-case control study	2019	Kempiński et al. [42]	Poland	Correlation between NAFLD and diverticulosis (*p* < 0.005; OR 1.65)	1058(211)	529(–)	529(–)	(–)	Retrospective case-control study	Very low
Association of obesity with colonic diverticulosis in women	2020	Peery et al. [41]	USA	Women (BMI > 30) and colonic diverticulosis (PR 1.48; 95% CI 1.08–2.04)	623(259)	274(124)	349(135)	54.2	Prospective study	Low
Risk factors for endoscopic severity of diverticular disease of the colon and its outcome: a real-life case–control study	2020	Tursi et al. [27]	Italy	Age > 70 (OR 1.158; 95% CI 1.067–1.256); BMI > 30 (OR 1.378; 95% CI 1.241–1.531); hypertension (OR 1.092; 95% CI 1.007–1.185); diabetes mellitus (OR 0.783, 95% CI 0.682–0.898); CRC (OR 0.354; 95% CI 0.280–0.448)	11,086(5635)	5968(2984)	5118(2651)	(–)	Retrospective case-control study	Low
Magnetic resonance imaging of diverticular disease and its association with adipose tissue compartments and constitutional risk factors in subjects from Western general population	2020	Storz et al. [58]	Germany	Advanced diverticular disease: BMI (OR 1.69; 95% CI 1.24–2.29); TAT (OR 1.76; 95% CI 1.29–2.41); VAT (OR 1.64; 95% CI 1.17–2.32); SAT (OR 1.77; 95% CI 1.29–2.43)	371(216)	216(95)	155(121)	56.2	Cross-sectional analysis	Low
Environmental and dietary risk factors for colonic diverticulosis and diverticulitis	2021	Lukosiene et al. [31]	Germany and Lithuania	Age (OR 1.079; 95% CI 1.06–1.1); obesity (OR 1.05; 95% CI 1.02–1.09)	1333(858)	635(424)	696(434)	62.4	Cross-sectional analysis	Low
Type 2 diabetes and risk of diverticular disease: a Danish cohort study	2022	Wittström et al. [28]	Denmark	Diabetes mellitus type 2 (HR 0.88, 95% CI 0.80–0.96); lower among pts > 5 years duration of diabetes (HR 0.76, 95% CI 0.67–0.87) than < 4.9 years duration	225,653(8527)	105,629(–)	120,024(–)	59.5	Observational cohort study	Moderate
Cardiovascular risk factors and physical fitness among subjects with asymptomatic colonic diverticulosis	2022	Ukashi et al. [29]	Israel	Age [50–59 (OR 2.57; 95% CI 1.52–4.34); 60–69 (OR 2.87; 95% CI 2.09–3.95); > 70 (OR 4.81; 95% CI 3.23–7.15)]; smoking (OR 1.27; 95% CI 1.05–1.55); hypertension (OR 1.27; 95% CI 1.03–1.56); obesity (OR 1.36; 95% CI 1.06–1.74); male (OR 1.29; 95% CI 1.02–1.64)	4586(799)	3406(644)	1180(155)	56.0	Cross-sectional analysis	Low
Age, alcohol, sex, and metabolic factors as risk factors for colonic diverticulosis	2022	Yan et al. [30]	China	Age > 60 (OR 2.149; 95% CI 1.511–3.057); male (OR 1.878; 95% CI 1.373–2.568); obesity (OR 1.446; 95% CI 1.100–1.902); alcohol (OR 1.518; 95% CI 1.213–1.901); hypertension (OR 1.454; 96% CI 1.181–1.789); hypertriglyceridemia (OR 1.287; 95% CI 1.032–1.607); hyperuricemia (OR 1.570; 96% CI 1.257–1.961)	6180(449)	4004(382)	2176(67)	48.1	Cross-sectional analysis	Low
Prevalence of and risk factors for incidental colonic diverticulosis	2022	Wlodarczyk et al. [32]	USA	BMI > 30 (OR 2.22; 95% CI 1.03–4.80); Hispanic ethnicity (OR 10.05; 95% CI 1.74–58.26); alcohol (OR 3.44; 95% CI 1.26–9.39) in pts > 40 years	359(156)	205(65)	154(91)	38.0	Cross-sectional analysis	Very low
Percentage of body fat is associated with increased risk of diverticulosis: a cross sectional study	2022	Shih et al. [40]	China	Highest quartile of percentage of body fat (OR 2.089; 95% CI 1.436–3.030); higher correlation in female vs male and in pts > 60	5557(346)	3141(243)	2416(103)	51.2	Cross-sectional analysis	Low

### Main findings

#### Diabetes mellitus

Study results on the relationship between diabetes mellitus and diverticular disease are inconsistent. However, data from Korea suggest that there is a clear correlation between diabetes and diverticulosis in the entire colon (OR 2.08; 95% CI 1.23–3.53) and right colon (OR 2.40; 95% CI 1.40–4.15), but not with left-sided diverticulosis [23]. Data from a Japanese study by Sakuta and Suzuki also suggested an association between diabetes and diverticular formation [24]. Braunschmid et al. present European data that also show a trend toward a higher incidence of diverticula in individuals diagnosed with diabetes [25]. In contrast, the studies by Kopylov et al. (OR 0.488; 95% CI 0.289–0.825) and Tursi et al. (OR 0.783, 95% CI 0.682–0.898) show a significantly lower number of diverticulosis patients in the diabetic collective [26, 27]. Wittström et al. also described similar results (HR 0.88, 95% CI 0.80–0.96), with longer disease duration associated with a further risk reduction for the occurrence of diverticulosis [28]. Korean data from Song et al. could not show any associations [18].

#### Hypertension

Regardless of ethnicity, patients with arterial hypertension had a higher prevalence of diverticulosis [18, 24, 27, 29, 30]. Yeo et al. found a correlation between diverticulosis and poorly controlled hypertension (OR 2.07, 95% CI 1.17–3.67), the absence of antihypertensive treatment (OR 1.73; 95% CI 1.06–2.83), and a significant overall link (OR 1.83; 95% CI 1.21–2.75). However, with proper adherence to treatment resulting in normal blood pressure, there was no significant difference compared to the control group. These findings build upon prior studies that have established an association between asymptomatic diverticulosis and hypertension [17]. The study conducted by Tursi et al. demonstrates a significant association between hypertension and the development of diverticula, supported by a substantial number of cases (OR 1.092; 95% CI 1.007–1.185) [27].

#### Obesity

Studies examining the association between diverticulosis and obesity showed consistent data—a higher BMI was associated with more colonic diverticula in several studies [26, 27, 29–32]. Comstock et al. demonstrated a positive association between obesity and diverticula with increasing BMI (OR 2.7; 95% CI 1.6–4.7) [33]. Similarly, data by Mashayekhi et al. showed an association of diverticulosis with increasing body weight (BMI 25–30 (OR 3.02; 95% CI 1.33–6.88); BMI > 30 (OR 4.43; 95% CI 1.88–10.49)) [34]. Furthermore, a higher BMI is also associated with a more severe form of diverticulosis (OR1.44; 95% CI 1.06–1.96) [35]. A study found that patients with diverticular formation had an increased accumulation of abdominal visceral and subcutaneous fat, as observed on an abdominal CT scan, even though there were no differences in BMI between the diverticulosis and control groups [36]. Nagata et al. confirmed this finding, which was also noted in patients with diverticulosis who had a BMI below 25, indicating a normal weight. Furthermore, the increased visceral fat volume presented differentially associated with diverticular location (right (OR 1.79; 95% CI 1.24–2.58); left (OR 2.30; 95% CI 1.31–4.03); bilateral (OR 2.99; 95% CI 1.75–5.11)) [37]. In addition to the association of diverticulosis with increased abdominal fat, individuals with higher waist circumferences also had a higher incidence of diverticular disease. This has been demonstrated in both a US cohort (OR 2.8; 95% CI 1.7–4.9) [33] and a Portuguese cohort (OR 2.129, 95% CI 1.005–4.510) [38]. In another study, a correlation was found between waist-to-hip ratio and diverticulosis (OR 1.035; 95% CI 1.000–1.070) [39]. The study conducted by Shih et al. emphasizes a particularly noticeable impact observed within the female group. They found a significant correlation between being in the highest quartile of body fat percentage and the occurrence of diverticula (OR 2.089; 95% CI 1.436–3.030) [40].

In addition to body measurements, several metabolic laboratory parameters were assessed, which included leptin, low molecular weight adiponectin, and the serum concentration of sRAGE (soluble receptor for advanced glycation end products) [33]. While increased leptin concentrations were associated with higher rates of diverticulosis (OR 2.4; 95% CI 1.4–3.9), low molecular weight adiponectin (OR 0.5; 95% CI 0.3–0.8) and sRAGE (OR 0.4; 95% CI 0.3–0.7) concentrations were inversely associated.

Peery et al. demonstrated a significantly increased risk of colonic diverticula in women with a BMI > 30 and age less than 60 years, although no correlation was found in the male study population. The cause of this phenomenon in general obesity and non-central obesity was attributed to increased testosterone concentration and decreased concentration of SHBG (sex hormone-binding globulin) and total estrogen. Premenopausal ovarian steroid hormones consequently could have a protective effect with regard to diverticular formation [41].

#### Fatty liver disease

Data on the association of diverticulosis and fatty liver disease are conflicting. While Bae et al. and Kempinski et al. showed a correlation between increasing levels of fatty liver and diverticulosis (moderate FLD (OR 2.238; 95% CI 1.026–4.882); severe FLD (OR 5.519; 95% CI 1.236–21.803)) [39, 42], Sahin et al. identified fatty liver disease as a negative predictor of diverticulosis (OR 0.529; 95% CI 0.323–0.866) [43]. Interestingly patients in the latter study were 65 years or older, while Bae et al. and Kempinski et al. included younger patients.

#### Metabolic syndrome (MS)

An association between metabolic syndrome and diverticulosis was only investigated in one study. Data from Teixeira et al. showed an association between metabolic syndrome and asymptomatic diverticulosis (OR 3.682, 95% CI 1.587–8.546) [38].

### Incidental findings

#### Age

The literature search found a strong link between patient age and the presence of diverticulosis. As patients get older, they have a higher likelihood of developing diverticula, regardless of their ethnicity or where the diverticula appear in the colon [18, 23, 25–27, 29–31, 35, 38, 44]. As shown by Korean studies performed by Song et al. and Lee et al., age is a risk factor for diverticulosis in the right and left sided colon [18, 23]. Data from Storz et al. and Song et al. indicate that patients with only left-sided diverticula are, on average, much older than patients with only right-sided diverticulosis [18, 35]. In addition, Storz et al. showed that the severity of diverticulosis increases with age (OR 2.24; 95% CI: 1.60–3.15) [35]. These findings were also confirmed in a study by Peery et al., in which increased age was associated with multiple diverticula (> 10) and larger or deeper diverticula in patients < 50 years compared with > 60 years. As already mentioned by this study, it is obvious that overall it is a progressive phenomenon by lifetime [44]. It is speculated that the correlation between age and increased incidence of diverticula might be due to changes in the structure of the colon [27].

#### Sex

Three of the included studies showed a significant clustering of diverticulosis patients in the male collective after multivariable analysis whereby the data of Kopylov et al. (OR 1.737; 95% CI 1.346–2.241) [26] were similar to those of Ukashi et al. (OR 1.29; 95% CI 1.02–1.64) [29] and Yan et al. (OR 1.878; 95% CI 1.373–2.568) [30]. In the majority of the included studies, no clear gender-dependent correlation could be found, in contrast to previously published studies [45].

#### Hypothyroidism

One study examined a possible association of hypothyroidism and diverticulosis. Patients with a medical history of hypothyroidism were found to have a higher prevalence of colonic diverticula (OR 2.403; 95% CI 1.303–4.431). Therapy adherence or cause of hormonal imbalance, however, was not further explored in this study [26].

#### Colon adenoma

Data on the occurrence of adenomas and asymptomatic diverticulosis were inconsistent. While Teixeira et al. and Tursi et al. had similar rates of adenoma in the diverticulosis and control group [27, 38], Lee et al. showed a significantly higher rate of adenomas in bilateral diverticulosis (OR 2.72; 95% CI 1.94–3.82) and proximal colonic diverticula (OR 2.36; 95% CI 1.66–3.38) [23].

#### Constipation and dietary habits

Studies did not find a clear association between dietary habits and the incidence of colonic diverticula [27, 31, 35]. The data published by Braunschmid et al. were also controversial with regard to the previously assumed correlation with a high-fiber diet [25]. In contrast, the increasing prevalence of diverticular formation in Asian regions has been attributed to the adoption of Western dietary habits in these areas [18, 23].

#### Alcohol

In the data from Korea presented by Song et al., alcohol shows a positive correlation with the occurrence of diverticula, as in other diseases of the digestive tract. With regard to drinking habits, only non-drinkers and drinkers were categorized, which is why there is a certain limitation in terms of significance [18]. The data collected by Nagata et al. also showed a significant correlation between alcohol consumption and diverticular formation, although this study also differentiated according to the amount of alcohol consumed. However, a dose-dependent enhancement of the effect was not found, although this is difficult to assess due to the small number of cases [37]. A correlation with alcohol without further definition of drinking quantity was also evident in data from Yan et al. (OR 1.518; 95% CI 1.213–1.901) [30] and Wlodarczyk et al. in patient over 40 years (OR 3.44; 95% CI 1.26–9.39) [32]. In contrast, the study by Bae et al. showed similar results in terms of alcohol consumption in control and diverticulosis groups and consequently no correlation [39].

#### Hyperlipidemia

Correlating with the severity of diverticulosis, more severe findings with multiple diverticula were observed in patients with higher LDL levels (OR 2.35; 95% CI 1.09–5.06) compared with the control group and milder manifestation [35]. In the data of Yan et al., there was also a correlation with hypertriglyceridemia (OR 1.287; 95% CI 1.032–1.607) [30].

## Discussion

Given the increasing global prevalence and its consequential economic impact, the prevention of diverticulosis has emerged as a crucial area of focus [3–5]. This systematic review specifically focuses on the metabolic risk factors associated with the development of asymptomatic colonic diverticula. Despite the inconsistent data, individual pieces of evidence suggest a link between diverticulosis and certain metabolic factors.

Regarding the anatomical distribution of diverticula, there are significant geographic and morphological differences between Asian and Western civilizations, as described earlier [1, 2]. Several of the included studies analyzed the location of diverticula in relation to potential causes or associations with metabolic components. The presented trends regarding hypothesized different genesis of right- or left-sided diverticula are currently difficult to generalize due to heterogeneous data and different study collectives. The predominantly right-sided localized diverticula in the Asian region are likely to follow a genetic cause, for which the WNT4, RHOU, and OAS1/3 genes may be important, as presented in the genome-wide association study by Choe et al. [46]. These could cause differences in the neurohumoral system as well as different morphology of the colonic wall between Asian and Caucasian populations [47]. Finally, it should be noted that a comparison, presuming this genetic variability, between right-sided diverticula formation in the Western and Asian populations could be problematic. Therefore, further large population-based studies considering diverticular localization are warranted.

Regarding hypertension, Yeo et al. found a correlation between diverticulosis and therapeutic intervention and therapy adherence in hypertensive patients, regardless of ethnicity, who had significant diverticulosis formation. No significant risk of diverticulosis was observed in patients with well-controlled hypertension and physiological blood pressure levels [17]. Earlier in this study, it was mentioned that the underlying cause of diverticula is still not well understood. However, it has been suggested that the sites where the vasa recta pass through the lamina muscularis may be susceptible to diverticula formation. This may be due to changes in the vessels and surrounding structures in this area [48]. Consistent with the positive correlation with patient age [18, 23, 25–27, 31, 35, 38, 44], the aging process of the vessels, favored by hypertension, results in endothelial dysfunction [49], remodeling of the extracellular matrix, calcification, and overall increased vascular stiffness [50]. Furthermore, it appears that micro-inflammatory processes in the vessels combined with hypertension may contribute to permanent vascular damage through fibrosis and apoptosis, although the exact mechanism is yet to be determined [51, 52]. These blood vessel changes lead to structural changes and further weaken the already vulnerable areas that tend to bulge. This suggests a link between elevated blood pressure levels and changes in the colon wall that contribute to diverticular formation. More research is needed to fully understand this potentially causal relationship.

As previously noted, advanced age is a major risk factor for the development of diverticula, regardless of ancestry and location of the diverticula [18, 23, 25–27, 31, 35, 38, 44]. According to the data of the included studies, more severe findings with multiple, deeper, and larger diverticula were observed with increasing age of the patients [35, 44]. In terms of left-sided diverticulosis, an older patient population was observed overall [18, 35]. Structural changes in the colon wall seem to be the cause, as already reported by Tursi et al. in their study [27]. As mentioned above, diverticula are formed preferentially in younger patients due to various connective tissue diseases. While degeneration of connective tissue in old age has been best studied in the skin, also because of its easy accessibility, there is data on degenerative changes in intestinal smooth muscle in animal models. These are caused by impaired signal transduction, although evidence for this in the human organism is unclear. Suggestive associations with weakening of the intestinal wall are also shown in animal models in relation to neurodegenerative age-related changes. In humans, however, there are few functional studies, although promising data showed a reduction in the proportion of intestinal nerves expressing choline acetyltransferase [53]. Peery et al. suggests that this is a progressive phenomenon over the lifetime [44]. Estimating the frequency of asymptomatic diverticulosis in a population is a challenging endeavor, particularly when it comes to younger patients who typically undergo colon-related investigations only when they exhibit symptoms. Consequently, although several studies have demonstrated an increasing prevalence of diverticulosis across all age groups, there exists a scarcity of direct evidence regarding its occurrence specifically among individuals under the age of 50 [3, 4].

With regard to diabetes mellitus, the studies show contrasting results. However, the data of Lee et al. seem to be particularly interesting, showing an association between the occurrence of diverticula in the right and the entire colon and diabetes, but no association with isolated left-sided diverticula [23]. Due to the absence of association in the data of Uri et al. [26] and Tursi et al. [27], a conclusive statement about a connection between diabetes and diverticular formation cannot be made on the basis of the available data. Wittström et al. suggest that the duration of the disease provides additional protection against diverticula [28]—one explanation for this counterintuitive finding could be lifestyle changes, which are a cornerstone of diabetes therapy.

As already established by the meta-analysis conducted by Wijarnpreecha et al., there is a clear association between diverticulosis and obesity [19, 34]. A more pronounced finding of diverticulosis in relation to body weight was also demonstrated [35]. Independent of BMI, there was an association between diverticular formation and measured subcutaneous and visceral fat accumulation; this association was also confirmed in normal-weight patients [37]. There was also an association with increasing waist circumference [33] and waist-to-hip ratio [39], although the pathophysiological background up to now remains unclear. Correlating with the severity of diverticulosis, elevated LDL levels were also detected in more severe forms [35]. In the female patient population, a correlation could be found in the younger patient population due to the increased testosterone concentration and decreased concentration of SHBG (sex hormone-binding globulin) and total estrogen in obesity. This suggests a protective effect regarding diverticulosis of premenopausal ovarian steroid hormones [41].

While no correlation between fatty liver disease and diverticulosis was shown in the older patient population [43], a correlation increasing with the degree of steatosis was shown, especially in younger patients [39, 42].

The hypothesized direct association between metabolic syndrome and diverticulosis was confirmed in only one of the included studies [38]. Ukashi et al. describes an increased 10-year risk of ischemic heart disease and stroke in patients between 50 and 69 with asymptomatic diverticulosis estimated by ASCVD risk score—suggesting a link between cardiometabolic risk factors and diverticulosis [29]. On the one hand, a causal relationship by inflammatory chronic processes or by alteration of the microbiome [54], on the other hand, the common risk factors as a cause would be conceivable. However, focused investigation regarding the correlation between diverticulosis and cardiometabolic risk factors is scarce and therefore definitely requires further investigation.

Finally, it should be added that in addition to possible effects of cardiometabolic risk factors on the development of diverticulosis, the reciprocal relationship of diverticulosis and cardiovascular risk factors could also be of interest. Thus, it would at least be conceivable that low-grade systemic inflammation is triggered or exacerbated by diverticulosis and leads to unfavorable cardiovascular outcomes. In this regard, however, we can only emphasize the potential relevance since numerous people have diverticula, and therefore, there may be a slight biological effect of diverticulosis on cardiovascular outcomes, which remain the leading cause of death worldwide. This calls for further investigation in future studies.

One of the strengths of the present work is that it is a systematic literature review, which provides a comprehensive and unbiased summary of all currently available knowledge. The predefined and rigorous methodology, including explicit criteria for study selection, data extraction, and quality assessment, minimizes the risk of bias. By including studies from diverse populations, settings, and methodologies, the results gain generalizability. The process of systematic review is transparent and well-documented, making it reproducible by third parties. Despite making every effort to avoid bias, there is, of course, a residual risk of overestimating results due to the non-publication of negative or inconclusive findings. The heterogeneity of the data, especially in comparison with the Asian population, where the location and possibly the origin of diverticula differ, makes it challenging to pool data and draw meaningful conclusions. The predominantly low quality of the available studies should be considered in the context of the conclusions.

## Conclusion

In summary, the data synthesis indicates a correlation between arterial hypertension, obesity, and fatty liver disease among younger patients. Nevertheless, it appears that the age of the patients and genetic factors are significant determinants in the development of diverticula. As for diabetes mellitus, the existing data is inconclusive and may necessitate further investigation, particularly concerning the location of the diverticula.

The present study highlights a potential correlation with cardiometabolic risk factors. Building upon these findings, it is imperative to conduct more focused research to gain a deeper understanding of how metabolic risk factors contribute to the development of asymptomatic diverticulosis. To achieve this, future investigations should consider exploring the specific mechanisms through which these risk factors influence diverticulosis onset and progression, as well as identifying potential preventive and therapeutic interventions aimed at mitigating this condition in individuals with cardiometabolic risk factors. Additionally, larger sample sizes and longer-term follow-up studies could provide valuable insights into the long-term implications and outcomes of asymptomatic diverticulosis in relation to these risk factors.

## Data Availability

A Prism checklist (2020) has been created and attached.
